# Eight-Week Training Cessation Suppresses Physiological Stress but Rapidly Impairs Health Metabolic Profiles and Aerobic Capacity in Elite Taekwondo Athletes

**DOI:** 10.1371/journal.pone.0160167

**Published:** 2016-07-27

**Authors:** Yi-Hung Liao, Yu-Chi Sung, Chun-Chung Chou, Chung-Yu Chen

**Affiliations:** 1 Department of Exercise and Health Science, National Taipei University of Nursing and Health Sciences, Taipei City, Taiwan; 2 Department of Chinese Martial Arts, Chinese Culture University, Taipei City, Taiwan; 3 Physical Education Office, National Taipei University of Technology, Taipei City, Taiwan; 4 Department of Exercise and Health Science, University of Taipei, Taipei City, Taiwan; Glasgow University, UNITED KINGDOM

## Abstract

Changes in an athlete’s physiological and health metabolic profiles after detraining have not been studied in elite Taekwondo (TKD) athletes. To enable a better understanding of these physiological changes to training cessation, this study examined the effects of 8-weeks detraining on the aerobic capacity, body composition, inflammatory status and health metabolic profile in elite TKD athletes. Sixteen elite TKD athletes (age: 21.0 ± 0.8 yrs, BMI: 22.4 ± 3.9 kg/m^2^; Mean ± SD; 11 males and 5 females) participated in this study. Physical activity level assessment using computerized physical activity logs was performed during the competitive preparation season (i.e. one-week before national competition) and at two week intervals throughout the detraining period. Participant aerobic capacity, body fat, and blood biomarkers were measured before and after detraining, and the blood biomarker analyses included leukocyte subpopulations, blood glucose, insulin, dehydroepiandrosterone-sulfate (DHEA-S), and cortisol. Eight-week detraining increased DHEA-S/cortisol ratio (+57.3%, *p* = 0.004), increased insulin/cortisol ratio (+59.9%, *p* = 0.004), reduced aerobic power (–2.43%, *p* = 0.043), increased body fat accumulation (body fat%: +21.3%, *p* < 0.001), decreased muscle mass (muscle mass%: –4.04%, *p* < 0.001), and elevated HOMA-IR (the biomarker of systemic insulin resistance; +34.2%, *p* = 0.006). The neutrophil-to-lymphocyte ratio (NLR), a systemic inflammatory index, increased by 48.2% (*p* = 0.005). The change in aerobic capacity was correlated with the increased fat mass (*r* = –0.429, *p* = 0.049) but not with muscle loss. An increase in the NLR was correlated to the changes in HOMA-IR (*r* = 0.44, *p* = 0.044) and aerobic capacity (*r* = –0.439, *p* = 0.045). We demonstrate that 8-week detraining suppresses physiological stress but rapidly results in declines in athletic performance and health metabolic profiles, including reduced aerobic capacity, increased body fat, muscle loss, insulin resistance development and elevated systemic inflammatory status in these young elite TKD athletes. The inflammation state was positively associated with insulin resistance development, fat mass, WHR (the index for central fat accumulation), and the decline in VO_2_max.

## Introduction

Taekwondo (TKD) is a traditional Korean martial art focused on the “way of kicking and punching”, initially designed for military combat and self-defense purposes. The Sydney Olympic Games introduced TKD as a sport in 2000 [1, 2]. TKD receives great attention from practitioners, national sport authorities and sport scientists. The TKD attack strategy focuses primarily on powerful kicking techniques [3, 4]. This combat approach is quite different from other Olympic combat sports such as boxing, wrestling and judo. TKD combat competition performance is closely associated with the athletes’ strength and conditioning, physiological characteristics, skills, tactical decisions and psychological status [5]. TKD requires unique physiological demands from various jumping and weight shifting kicking exercises, requiring cardiorespiratory fitness, low body fat level, high anaerobic power, rapid movement skills, powerful jumping and spinning, punching and overall agility [1, 2, 6, 7]. Intensified physical and technical training are therefore important for elite TKD athletes to enhance their physical performance during the competitive preparation season. Optimizing and sustaining training outcomes for excellent competitive performance is very important and time sensitive.

Periodic training programs for competitive sports, including TKD, consist primarily of a competitive preparation season with intensified physical and sport-specific skill training, main competitive events and the off-season recovery/transition period [8, 9]. During the off-season period the regular strength and conditioning training load is decreased or completely ceased. Previous evidence revealed that following a 3–8 week training cessation, endurance capacity declined [10–12], insulin resistance developed [13, 14], body fat increased [10, 13, 14], muscular strength and power decreased and muscle mass loss [10, 12, 15] was observed in athletes from varied sporting disciplines. Detraining induced decrease in metabolic functions, particularly insulin sensitivity, can therefore weaken the post-exercise recovery process (e.g. glycogen replenishment, muscle protein synthesis, etc.), training adaptations and athletic performance [16–18]. Published evidence also revealed that visceral fat accumulation is strongly associated with increased systemic inflammatory responses [19–22] and the declines in insulin sensitivity [22]. More importantly, based upon a case study on Olympic champion rowers [23], the training adaptation losses following short-term detraining requires twice the time to recapture the competitive season performance level. Recent evidence suggests that substantial time may be needed to help athletes regain their competitive edge following detraining.

Most studies focus on performance training, field-testing methods, physiological responses during competition match, or sport injuries in TKD athletes [1, 2, 6, 24]. None of these studies analyzed the impacts of detraining on physical health and metabolic profiles in the TKD population. With this in mind, a comprehensive understanding of TKD sport-specific physiological demands, including training and detraining periods, is critical to the development of appropriate TKD periodic training programs. There are two important unanswered issues in this popular combat sport: (1) whether the aerobic capacity and optimal body composition last after short-term training cessation and (2) whether detraining impairs health metabolic profiles (e.g. insulin sensitivity, anabolic/catabolic balance, etc.). Here we hypothesized that a short-term detraining would rapidly impair aerobic capacity, body composition and health metabolic profiles in elite TKD athletes. The purpose of this study is therefore to investigate the 8-week detraining effects on physiological stress status, aerobic capacity, body composition, inflammatory state and health metabolic profiles in Division I elite TKD athletes.

## Methods and Materials

### Participants and ethics statement

The Institute Review Board (IRB) of the University of Taipei reviewed and approved this study prior to participant recruitment. This study conducted all procedures according to the principles expressed in the Declaration of Helsinki. Sixteen elite TKD athletes with a minimum of 8 years competitive experience in the sport (age: 21.0 ± 0.2 years, body mass: 68.5 ± 3.2 kg, height: 174.6 ± 1.7 cm; 11 males and 5 females) voluntarily participated in this study. All participating athletes were black belt holders and classified in the national Division I category. According to the World TKD Federation (WTF) Olympic weight classes, the distribution of participating athletes was as follows. Male participants (n = 11): <58 kg (n = 1), 58–68 kg (n = 4), 68–80 kg (n = 5), >80 kg (n = 1); female participants (n = 5): <49 kg (n = 1), 49–57 kg (n = 1), 57–67 kg (n = 1), >67 kg (n = 2). The athletes underwent an intensive periodic TKD-specific training program (10 training sessions per week; 14–15 hours per week) during the competitive preparation season, including 2–2.5 hours of aerobic training, 7.5–8 hours of TKD-specific tactical and skill training, 2.5 hours of strength and conditioning training and 2 hours of stretch and flexibility training. This training program is designed to prepare participants for the national annual TKD competition. All participating athletes practiced with this training program for at least 8 weeks before the national event. All participating athletes completed a written informed consent and self-screening health questionnaire prior to these experiments. Participants were free of hypertension, dyslipidemia, musculoskeletal injuries, and cardiovascular/metabolic disorders.

### Experimental design

**Fig 1:**
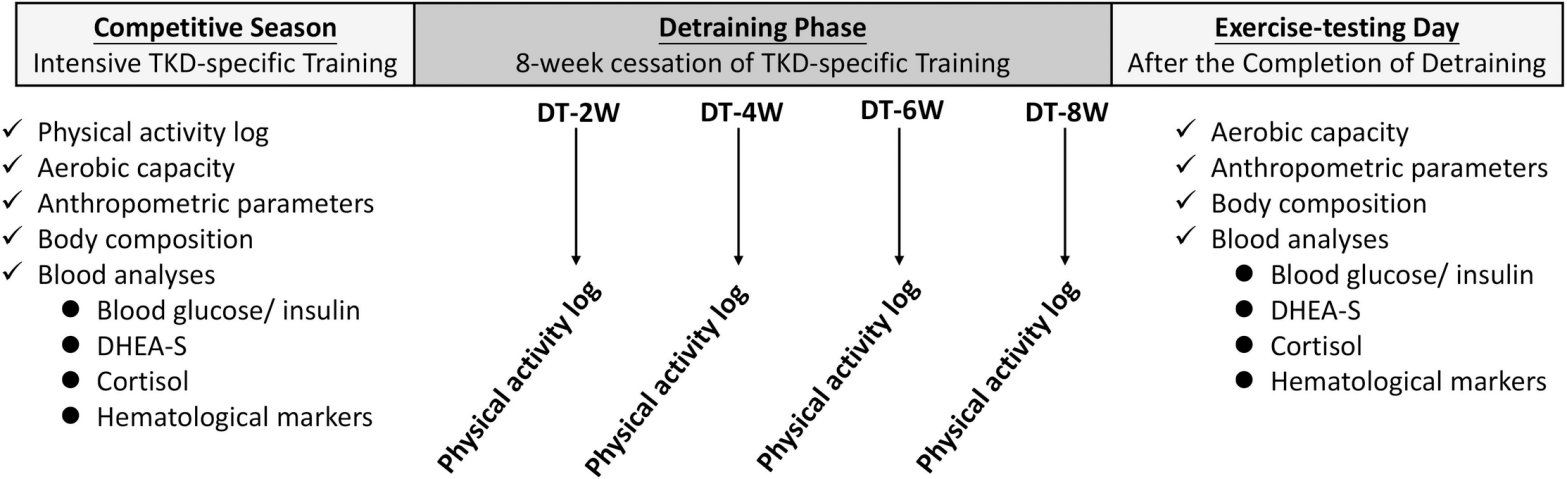
Time line of experimental design.

This study consisted of three consecutive phases: 1) competitive preparation season with intensive regular TKD-specific training (i.e. one-week before national annual competition; TKD-RT), 2) detraining phase: 8-week cessation of regular sport-specific training, and 3) the exercise-testing day immediately after the completion of detraining (Fig 1). We measured the participant’s initial physical activity status, aerobic capacity, anthropometric parameters, body composition during the competitive preparation season. We collected overnight fasting (12h) venous blood samples for further biochemical analyses. Prior to the venous blood sample collection, all participating athletes refrained from any form of exercise training or vigorous physical activity for 2 days to control possible confounding factors influencing blood biomarker assessments. The participating athletes thereafter undertook an 8-week detraining intervention to minimize the regular training load. Subjects were restricted from any form of exercise training or vigorous physical activity. To ensure good dietary pattern control throughout the detraining period we provided detailed nutritional guidelines to all athletes as previously described [12]. The subjects were instructed to consume food in accordance with the following compositions: carbohydrate (~60%), protein (10–15%), and fat (15–25%) based on their individual daily energy requirements. They were also asked to periodically maintain computerized physical activity logs (three-day physical activity log, 3-d PAL) during the competitive preparation season and detraining period (week 2, week 4, week 6, and week 8) [25]. All measurements and venous blood samples were collected as previously described to compare the differences in these parameters before and after detraining.

### 20-meter shuttle run test and VO_2max_ assessment

A 20-meter shuttle run test introduced by Leger and Mercier determined the subject’s maximal aerobic capacity [26]. A prerecorded tape emitted a sound signal to guide athletes to follow the running pace. Each athlete had to run between 2 lines spaced 20 meters apart at a pace set by the sound signals. The sound signal frequency started at 8.5 km/h and increased 0.5 km/h each minute. The signal pace kept increasing until the athlete could no longer follow the pace. The predicted maximal oxygen uptake was calculated as follows: (VO_2_max; ml/kg/min) = 31.025 + 3.238 × speed (km/h)– 3.248 × Age (yrs) + 0.1536 × speed (km/h) × Age (yrs).

### Anthropometric measurements and body compositions

All anthropometric measurements were performed after overnight fasting (12 h). All instruments were calibrated before the test. A non-stretchable tape measure was used to take hip and waist circumferences. The obtained values determined the waist-to-hip ratio (WHR). A bio-impendence body composition analyzer (InBody 720, InBody Co., Ltd, Seoul, Korea) determined the body fat (% BF) percentage and other body composition values [27].

### Analyses of hematological parameters and blood biomarkers

Whole blood samples were collected in tubes containing a clot activator with gel separator or anti-clotting reagent (ethylenediaminetetraacetic acid, EDTA). A portable glucose monitoring system (One Touch Ultra 2; LifeScan Inc., Milpitas, CA) measured the subject’s fasting blood glucose concentration. EDTA-treated whole blood samples determined the hematological parameters, including total white blood cells (WBC), neutrophils (Neu), and lymphocyte (Lym) using an automated hematology analyzer (Sysmex XT-2000, Sysmex Corp., Kobe, Japan). The remaining whole blood samples collected in tubes with clot activator were centrifuged at 3,000 ×g for 10 min (4°C). The serum from each blood sample determined the circulating levels of insulin, cortisol, dehydroepiandrosterone-sulfate (DHEA-S). The enzyme linked immunosorbent assay method (ELISA) with commercial available kits was used to determine the insulin serum levels (intra-assay CV% = 3.35%; #10-1113-01, Mercodia AB, Uppsala, Sweden), cortisol (intra-assay CV% = 8.35%; #500360, Cayman Chemical Co., Ann Arbor, MI, USA), and DHEA-S (intra-assay CV% = 5.43%; #IB79121, IBL Inc., Hamburg, Germany) in accordance with the manufactures’ instructions.

### Measurements of Insulin Sensitivity

We used an indirect index, the homeostatic model assessment of insulin resistance index (HOMA-IR index) [28], for the insulin resistance (IR) assessment. HOMA-IR was calculated using glucose and insulin fasting levels to determine the insulin resistance severity, HOMA-IR = [Glucose (mM) × insulin (mU/L)/22.5].

### Statistical Analysis

All data were analyzed and graphed using SPSS 16.0 software (SPSS, Chicago, IL, USA) and GraphPad Prism 5.0 (GraphPad software Inc., La Jolla, CA, USA), respectively. The Shapiro-Wilk normality test provided by SPSS 16.0 software analyzed the normality of all subject variables. The differences in anthropometric measurements, body composition parameters, aerobic capacity, insulin sensitivity, hormonal concentrations, and hematological profiles before and after 8-weeks detraining were compared using a paired *t-test*. Prior to performing the pair*-t* test, HOMA-IR, body fat %, muscle mass %, NLR, insulin, blood glucose, and DHEA/cortisol ratio (D/C ratio) variables were logarithmically transformed for the later statistical comparisons because of the skewed data distribution. Statistical comparisons were adjusted and corrected using the Bonferroni adjustment. One-way analysis of variance (ANOVA) with repeated measures compared the changes in physical activity level throughout the detraining period. Pearson correlation analyses evaluated the relationships among the percent changes (Δ% between pre- and post-detraining) in the measured parameters. All data were presented as mean ± standard error of mean (*Mean ± S*.*E*.*M*.), and the alpha level was set at 0.05 (*p* < .05) for statistical difference for all comparisons.

## Results

### Physical activity and endurance exercise capapcity

**Fig 2:**
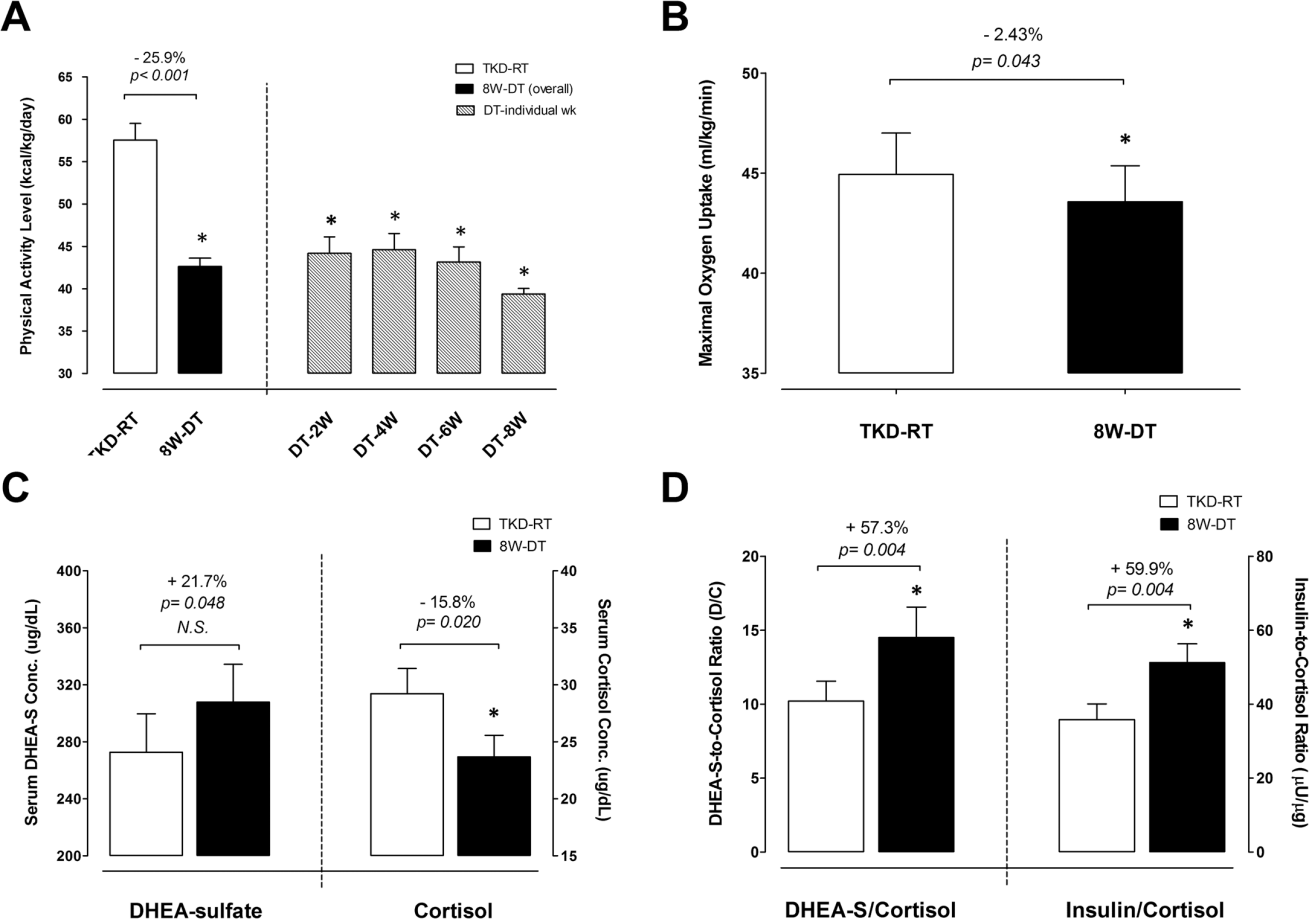
Changes in physical activity status, aerobic capacity and anabolic/catabolic hormonal concentrations in response to 8-weeks detraining. (A) The changes in physical activity levels, (B) exhanustive shuttle running distance and maximal oxygen consumption, (C) serum DHEA-S and cortisol concentrations, and (D) DHEA-S-to-cortisol ratio (D/C ratio) and insulin-to-cortisol ratio (I/C ratio) were measured during competitive preparation season with intensified TKD training and after an 8-week cessation of TKD training. TKD-RT: regular Taekwondo-specific training. 8W-DT: cessation of TKD training for 8 weeks. Values are expressed as *Mean ± S*.*E*.*M*. * Significant difference between TKD-RT and 8-week DT (*p* < 0.05).

A 3-day computerized recall log assessed the subject’s physical activity level during the TKD competitive preparation season (TKD regular training, TKD-RT) and detraining phase (8W-DT). The results are shown in Fig 2A. The overall average physical activity level during DT (42.6 ± 1.0 kcal/kg/day) was approximately ~25.9% less than the average value during TKD-RT (57.5 ± 2.0 kcal/kg/day). The physical activity levels throughout the detraining phase were significantly lower compared with the competitive preparation season (*p* < 0.001). Fig 2B shows the shuttle run distance and estimated maximal oxygen uptake during the competitive preparation season and after 8-weeks of detraining. The estimated maximal oxygen uptake decreased significantly by ~2.43% after 8-weeks detraining (*p* = 0.043) compared with the competitive preparation season.

### Responses of anabolic and catabolic hormones to long-term detraining

Fig 2C displays the fasting serum DHEA-S and cortisol levels. The serum DHEA-S level was higher after 8-weeks detraining than during the competitive preparation season with intensified training (+21.7%, *p* = 0.048). The increased DHEA-S did not reach statistical difference after Bonferroni adjustment. Conversely, the serum cortisol level was significantly lower after 8-weeks detraining (–7.6%, *p* = 0.020) compared with the competitive preparation season. The DHEA-S-to-cortisol ratio (D/C ratio) and insulin-to-cortisol level (I/C ratio) are shown in Fig 2D. After 8-weeks detraining, the D/C ratio and I/C ratio increased significantly by approximately ~57.3% and ~59.9% above the competitive preparation season level (D/C ratio: *p* = 0.004; I/C ratio: *p* = 0.004), respectively.

### Anthropometric measurements and body compositions

**Fig 3:**
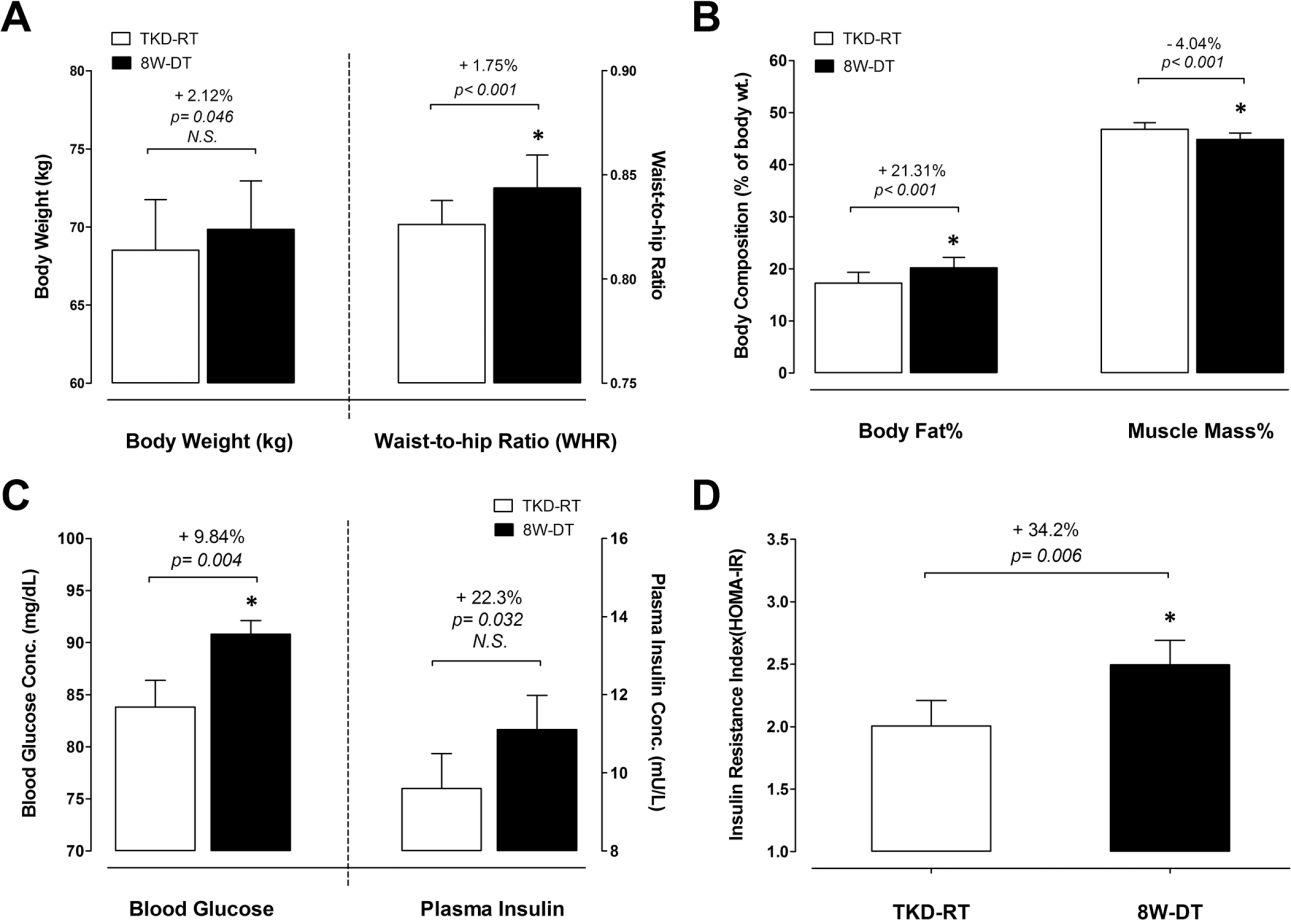
Changes in anthropometric measurements and insulin sensitivity in response to 8-weeks detraining. (A) The changes of body weight and waist-to-hip ratio (WHR), (B) body composition, (C) fasting blood glucose and insulin concentrations, and (D) HOMA-IR insulin resistance index were measured during competitive preparation season with intensified TKD training and after an 8-week cessation of TKD training. TKD-RT: regular Taekwondo-specific training. 8W-DT: cessation of TKD training for 8 weeks. Values are expressed as *Mean ± S*.*E*.*M*. * Significant difference between TKD-RT and 8-week DT (*p* < 0.05).

Fig 3A and 3B show the anthropometric measurements, including body weight (BW) and waist-to-hip ratio (WHR), percentage of body fat (% BF), and percentage of muscle mass (% MM) during the competitive preparation season and after 8-weeks detraining. After 8-weeks detraining, the WHR increased significantly by +1.75% (*p* < 0.001), whereas the BW showed only an approached significant increase by 2.12% (*p* = 0.046; non-statistical differences with Bonferroni adjustment). Detraining resulted in significant increases in % BF (+21.3%, *p* < 0.001) and significant decreases in % MM (–4.04%, *p* < 0.001).

### Glycemic control and whole-body insulin sensitivity

Fasting glucose and insulin levels measured during the competitive preparation season and after 8-weeks detraining are shown in Fig 3C. The fasting blood glucose concentrations increased significantly by ~9.84% after 8-weeks detraining (*p* = 0.004). The increase in plasma insulin level after 8-weeks detraining approached the significance level (+22.3%, *p* = 0.032; non-statistical differences with Bonferroni adjustment). The HOMA-IR index (Fig 3D) calculated insulin resistance. The HOMA-IR value was 2.0 ± 0.2 during the competitive preparation season, but the values elevated significantly to 2.5 ± 0.2 after 8-weeks detraining (+34.2%, *p* = 0.006).

### Hematological profiles and inflammatory indices

**Fig 4:**
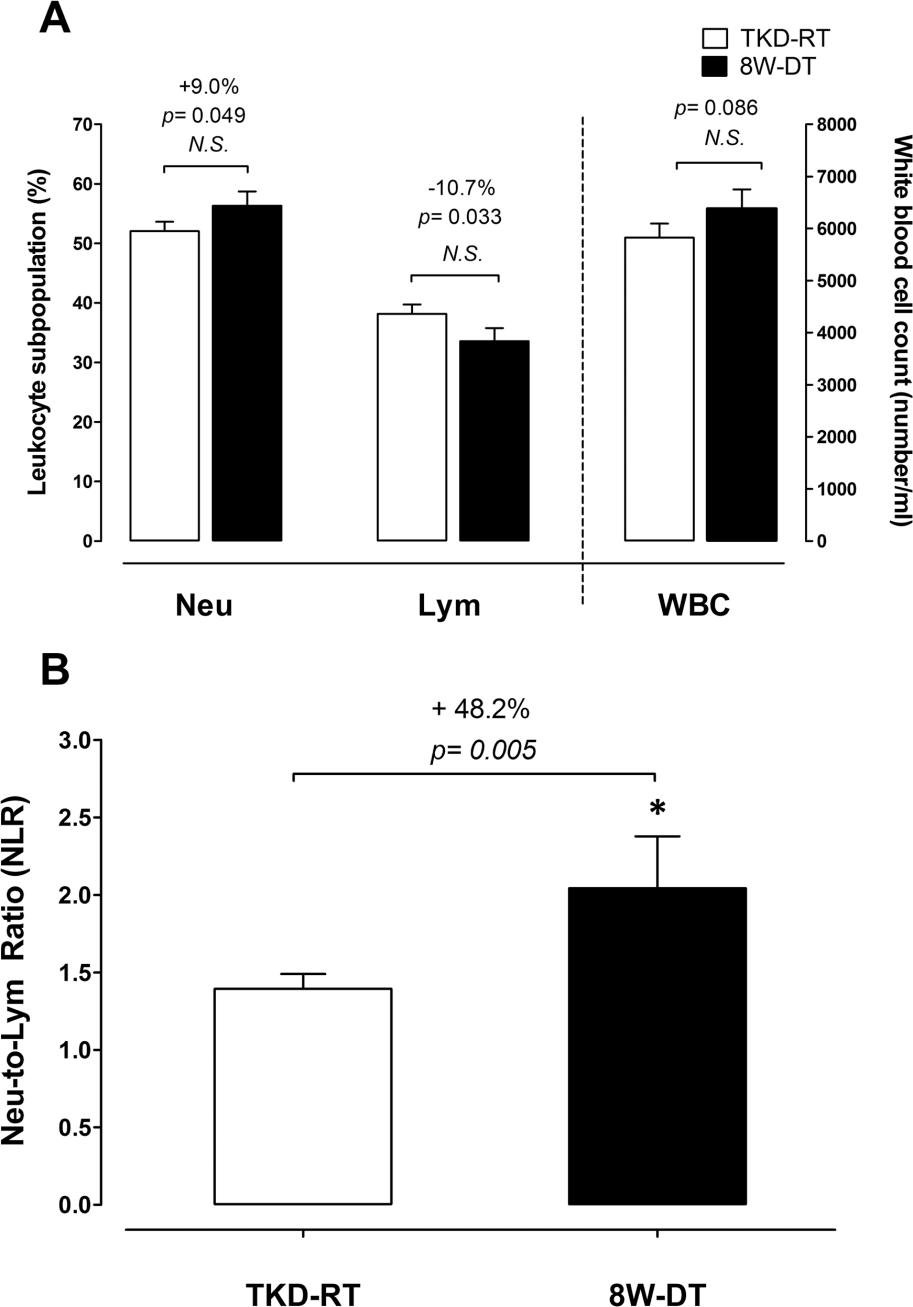
Changes in hematological parameters and systemic inflammation biomarkers. (A) The changes in total white blood cell count and leukocytes subpopulations (neutrophils and lymphocytes) and (B) systemic inflammation marker neutrophils-to-lymphocyte ratio (NLR) were measured during the competitive preparation season with intensified TKD training and after an 8-week cessation of TKD training. TKD-RT: regular Taekwondo-specific training. 8W-DT: cessation of TKD training for 8 weeks. Values are expressed as *Mean ± S*.*E*.*M*. * Significant difference between TKD-RT and 8-week DT (*p* < 0.05).

The hematological profiles and inflammatory indics are shown in Fig 4. Fig 4A displays the hematological profiles. No significant changes were observed in total white blood cell numbers between TKD-RT and 8W-DT phases (*p* = 0.086). Detraining raised the neutrophil counts by +9.0% (*p* = 0.049; non-statistical differences with Bonferroni adjustment) and decreased the lymphocyte count by 10.7% (*p* = 0.033; non-statistical differences with Bonferroni adjustment). Fig 4B displays the neutrophil-to-lymphocyte ratio (NLR), which represents the severity of inflammatory status. Compared with the baseline during the competitive preparation season, 8-weeks detraining significantly increased NLR by ~48.2% (*p* = 0.005).

### Correlation Analyses

We determined whether there were any relationships among the percent changes (Δ%) in aerobic power (VO_2_max), the insulin resistance index (HOMA-IR), body fat accumulation (BF mass and WHR), muscle mass, or systemic inflammation biomarker (Neu-to-Lym ratio, NLR). We found that the Δ% body fat mass showed significant correlations with Δ% aerobic power (*r* = –0.429, *p* = 0.049) and Δ% exhaustive run distance (*r* = –0.432, *p* = 0.047) after detraining. Moreover, the Δ% NLR showed remarkable relationships with Δ% HOMA-IR (*r* = 0.44, *p* = 0.044), Δ% body fat % (*r* = 0.51, *p* = 0.022), Δ% body fat mass (*r* = 0.63, *p* = 0.005), Δ% WHR (*r* = 0.716, *p* = 0.001), and Δ% VO_2_max (*r* = –0.439, *p* = 0.045). However, no significant correlations were observed between Δ% VO_2_max after detraining and Δ% HOMA-IR (*r* = 0.118; *p* = .331) or Δ% muscle mass (*r* = 0.343; *p* = .097) levels in response to training cessation.

## Discussion

To our knowledge, this is the first study investigating the detraining effects on sport performance-related properties in elite TKD athletes. The athlete parameters investigated included aerobic power, anabolic/catabolic hormone balance (D/C and I/C ratios), body composition, inflammatory status and metabolic profiles. The primary findings of this study were that 8-week detraining suppresses physiological stress but rapidly results in reduced aerobic capacity, increased body fat and muscle mass loss in young elite TKD athletes. The increase in body fat mass after detraining closely correlated to the reduced aerobic capacity. Importantly, insulin resistance (HOMA-IR) markedly elevated by ~34% after TKD-specific training cessation, and the development of insulin resistance was closely associated with an increase in body fat mass and systemic inflammatory status. Here we provide new evidence that an abrupt TKD training cessation negatively affects aerobic power and optimal body composition but also accounts for the marked increase in inflammatory status and the subsequent development of insulin resistance in these elite TKD athletes.

We observed that 8-weeks TKD-specific training cessation markedly decreased elite athletes’ physical activity level by ~26% below the competitive preparation season level. The index for physiological stress and anabolic capacity, evaluated by the ratios of anabolic hormones (i.e. DHEA-S and insulin) to catabolic hormone (cortisol), significantly elevated by approximately ~57–60% after training cessation (D/C and I/C ratios; Fig 2D). Although the DHEA-S level during the TKD regular training period was about ~21% lower than that after detraining, the difference only approached statistical significance. Our results, at least in part, confirmed previous findings that heavy training or external stress elicits greater physiological stress, thereby reducing circulating DHEA-S level [29–31] and systemic anabolic capacity (↓D/C ratio) [32]. Moreover, the gradual decline in circulating DHEA-S level has been reported during an intense high-intensity training program [33]. Based upon these previous findings [29, 30, 32, 33], we speculate that the dramatic decline in training load during detraining might account for the decreased physiological stress and thereby preserve the DHEA-S circulating level compared to the competitive preparation season. Of note, similar to the D/C ratio, an increased I/C ratio after detraining has also been suggested to exert an increased "anabolic drive" [34], thus our current results further validate the reduced physiological stress after short-term detraining. The increase in DHEA-S level after training cessation may directly reflect the declines in training load and physiological stress. Together with our and previous findings, the physiological challenge was substantially suppressed during the detraining period compared to that during the competitive preparation season.

Official TKD competition is characterized by short bouts with near maximal-intensity movements separated by less intense movements (2 min × 3 rounds) with short intervals between matches (1 min) [2]. Greater reliance on the anaerobic system occurs in this combat sport. However, several recent studies revealed that the aerobic metabolic system plays a crucial role in sustaining high-intensity activity and facilitating recovery between consecutive bouts during competitive events [35–39]. For all-out exercise with short recovery periods, higher aerobic capacity has been demonstrated in achieving greater recovery capacity after maximal exercise [40]. Detraining negative impact on endurance athlete aerobic power is well documented [10, 12]. The decline in aerobic power from training cessation is the result of the rapid decrease in muscular capillary density, oxygen delivery capacity, and mitochondrial respiratory functions [11, 41]. Here we observed that a reduction in training volume accompanies a slight but significant decline in VO_2max_ by approximately ~2.43% in these elite TKD athletes (Fig 2B). Previous evidence revealed that 2–8 weeks of detraining in highly trained endurance athletes [10, 11] and soccer players [12] brought about a 6–20% reduction in VO_2max_. The discrepancy for the magnitude of decreased VO_2max_ between our and previous studies could be associated with (1) the difference in sport discipline and training modes, (2) the duration of training cessation and (3) the initial cardiorespiratory fitness level. The decline in aerobic capacity from detraining could be the result from the decrease in muscular capillary density, oxygen delivery capacity and mitochondrial functions [11, 41]. We did not measure the muscular properties stated above, but observed a clear decrease in muscle percentage (↓4.04%), suggesting that a clear morphological and functional change could occur in muscle tissue. Our data demonstrated that the substantial decrease in training volume for 8 weeks was sufficient to impair aerobic capacity for this type of elite athlete. More retraining time is necessary to regain the athletes’ competitive edge.

The profiles of athletes competing at the international level mostly show low body fat percentage and moderate to high levels of aerobic power [2, 7, 42], suggesting the optimal body composition may be crucial to maintaining high performance levels during competition. For example, the winners competing at Olympic and national levels generally tended to be classified with less body fat (ranging from 7.3 to 13.2%), higher aerobic capacity, explosive power and agility [2, 7, 42]. However, detraining slightly increased body weight (↑2.12%) but significantly enlarged fat mass accumulation (↑21.3%) in these TKD athletes. More importantly, we found that there was a significant correlation between changes in body fat and aerobic capacity after detraining (*r* = –0.429). These results support previous findings of better aerobic capacity along with lower percent body fat in experienced TKD practitioners [43]. The lower body fat provides a lower weight-to-strength ratio (WSR) [44], which could yield a greater advantage in rapid acceleration and deceleration when performing combat movements. In addition, the suppressed physiological stress reflected by higher D/C ratio after detraining possibly accounts for the greater potential for energy substrate storage in adipose tissue but not in muscle tissue, thereby facilitating the deleterious changes in body composition and aerobic capacity. Our results demonstrate the adverse effects of short-term detraining on increased fat mass with musculature loss might diminish aerobic power and competitive advantage in elite TKD athletes.

For athletes who perform sports that involve near all-out exercise and short rest intervals, rapid recovery is important in preserving the subsequent sport performance. Insulin plays a primary role in the recovery process, including facilitating glycogen replenishment and protein resysnthesis in exercised muscle [16, 18]. Here we found that the degree of insulin resistance increased remarkably by ~34% (HOMA-IR increased from 2.00 to 2.49) after detraining. Note also that a HOMA-IR value of 2.77 is the clinical threshold for insulin resistance in metabolically healthy persons [45]. Intriguingly, based upon our results, only 8-weeks detraining was capable of rapidly decreasing insulin sensitivity toward the threshold for insulin resistance in these elite TKD athletes. Consistently, evidence from endurance athletes reveals that 3–8 weeks of detraining decreases insulin sensitivity and increases body fat [10, 13, 14]. The influence of visceral fat accumulation on impairing insulin sensitivity has close connections with the levels of adipose tissue-derived pro-inflammatory hormones, such as tumor necrosis factor-α (TNF-α), interleukin-6 (IL-6), and leptin [46–48]. We also observed a significant increase in the visceral fat mass as reflected by increased waist-to-hip ratio (WHR) after detraining, confirming the role visceral fat in the impaired insulin sensitivity in these TKD athletes.

One striking finding of this study is that an 8-week detraining strikingly elevated neutrophils-to-lymphocyte ratio (NLR), an inflammatory biomarker highly correlated to the circulating level of C-reactive protein [49], by ~48% above that during intensified TKD training (Fig 4B). Regular endurance training has been shown to reduce the pro-inflammatory cytokine TNF-α circulating level [50, 51], suggesting an anti-inflammatory effect from regular training. Of interest, Gill et al. (2003) reported that the anti-inflammatory effect could be diminished rapidly with exercise cessation in endurance-trained men [52], which is in agreement with our present finding that NLR increased with detraining. We also observed that NLR was strongly associated with HOMA-IR, body fat mass, WHR and the decline in VO_2_max. Our data provides evidence that detraining resulted in the accumulation of visceral fat, systemic inflammation and rapid development of insulin resistance in these elite TKD athletes, which might weaken training adaptation and recovery capacity. Future research is encouraged to determine the causal relationships among reduced physical activity, fat accumulation, systemic inflammatory responses and exercise performance in combat sports athletes.

### Practical Applications

For combat sports, a comprehensive understanding the changes of physiological and metabolic properties in response to short-term detraining is critical for coaches and sports scientists to develop practical interventions, such as specific training program, dietary manipulation, or ergogenic supplements, for preventing the rapid loss of training adaptions. The present study provides the evidence that an 8-week cessation of regular TKD training rapidly leads to markedly reduced aerobic capacity, negative alterations in body composition, reduced metabolic functions, and development of systemic inflammation in young TKD athletes. Our findings therefore provide fundamental knowledge not only for the competitive combat sport athletes who must experience a periodic training program but also for the coaches/team scientists who are responsible for athletic training.

## Conclusions

We demonstrated that, even in young elite TKD athletes, 8-weeks detraining suppress physiological stress and rapidly results in reduced aerobic capacity, induced body fat accumulation, muscle mass loss, impaired insulin sensitivity, and the rise of systemic inflammatory biomarker. We reported that the rise in systemic inflammatory state is significantly associated with insulin resistance, body fat mass, WHR and the decline in VO_2_max. This suggests that the adverse effects of detraining on the aerobic capacity and metabolic health profiles might be the result of increased visceral fat accumulation, thereby rapidly facilitating the development of systemic inflammation and deleterious changes toward insulin resistance development in these elite TKD athletes. Our present findings are also important for both coaching science and athletic health management professionals in developing practical interventions to eliminate the adverse impacts of detraining in this special population.
